# Two new species of the genus *Xya* Latreille, 1809 (Orthoptera, Tridactyloidea, Tridactylidae) from Yunnan with a key to all *Xya* species in China

**DOI:** 10.3897/zookeys.947.51067

**Published:** 2020-07-08

**Authors:** Chengquan Cao, Hua Rong, Hassan Naveed

**Affiliations:** 1 College of Life Science, Leshan Normal University, Leshan, Sichuan 614004, China Leshan Normal University Leshan China

**Keywords:** key, new species, Orthoptera, pygmy mole cricket, taxonomy, Tridactylidae, *
Xya
*

## Abstract

This contribution to the taxonomy of *Xya* Latreille, 1809 (Orthoptera, Tridactyloidea, Tridactylidae) adds descriptions and photographic illustrations of two new species: *Xya xishangbanna***sp. nov.** and *Xya yunnanensis***sp. nov.** from Xishuangbanna, Yunnan Province, China. *Xya xishangbanna***sp. nov.** can be diagnosed by the shiny dark brown hind femora, and the epiproct with a shallow bottom of the middle “v-shaped” crack in the upper part and straight sides; *Xya yunnanensis***sp. nov.** can be diagnosed by the compound eye bearing no narrow band along the inner margin, and the epiproct with the bottom of the side edge with a sharply angled protrusion and a narrow lower anchor-shaped base less than 1/2 the width of the upper one. Distributional information and bionomics for these two new species and photos for the habitat are given. A key to all Chinese species of *Xya* is provided.

## Introduction

The pygmy mole cricket genus *Xya* (Orthoptera, Tridactyloidea, Tridactylidae) was established by Latreille in 1809 with *Tridactylus variegatus* as its type species. The genus *Xya* Latreille, 1809 contains 59 described species worldwide, of which about 19 species are known to occur in Asia. According to the online Orthoptera Species File (http://orthoptera.speciesfile.org/HomePage/Orthoptera/HomePage.aspx, accessed 6 April 2018) the nine species that have been reported in China are: *Xya japonica* (Haan, 1844); *Xya nitobei* (Shiraki, 1911); *Xya manchurei* Shiraki, 1936; *Xya apicicornis* (Chopard, 1928); *Xya riparia* (Saussure, 1877); *Xya leshanensis* Cao, Shi & Hu, 2017; *Xya shandongensis* Zhang, Yin & Yin, 2018; *Xya sichuanensis* Cao, Shi & Yin, 2018 and *Xya fujianensis* Cao, Chen & Yin, 2020 (Latreille 1809; Haan 1844; Walker 1871; Saussure 1877, 1896; Brunner von Wattenwyl 1893; Bolívar 1900 (1899); Shiraki 1911; Chopard 1928, 1936, 1968; Bey-Bienko 1967; Günther 1974, 1980, 1995; Ingrisch 1987; Yin et al. 1996; Murai 2005; Yin et al. 2013; Heads and Hollier 2016; Kuravova and Kocarek 2016; Cao et al. 2017; Zhang et al. 2017; Cigliano et al. 2018; Cao et al. 2018 and Cao et al. 2020)

During an ongoing study of pygmy mole crickets, we collected a series of specimens belonging to the genus *Xya*, described two new species, namely *Xya xishangbanna* sp. nov. and *Xya yunnanensis* sp. nov., and provide a key to all the Chinese *Xya* species.

## Material and methods

All the jumping pygmy mole cricket specimens examined in the present study were collected by a small patented appliance (Cao et al. 2015) with high collection efficiency. Photos of the habitat were taken by a Canon camera (EOS 100D). After killed in a poison bottle with diethyl ether and the body postures arranged, specimens were examined using an Olympus SZX9 stereomicroscope, and habitus photographs and measurements were taken using a microscopic LY-WN system. All pictures were then processed using Photoshop CS6 software.

All examined specimens are deposited in the Leshan Normal University, Leshan, Sichuan Province, China.

## Taxonomy

### Xya xishangbanna sp. nov.

Taxon classificationAnimaliaOrthopteraTridactylidae

B114FDF8-C416-58DE-AB59-096B29707BDF

http://zoobank.org/2BA1572A-C865-4DFE-81DF-91CE1E936591

Figures 1–7

#### Type material.

***Holotype***: China • ♂; Yunnan Province, Xishuangbanna, Mengla County, Wuxiangguangchang; 21.92N, 101.11E; 21–24 Mar. 2019; leg. Chao Tong and Shenzhi Chen.

#### Paratype.

China • 1♀, same data as holotype.

**Figures 1–7.**
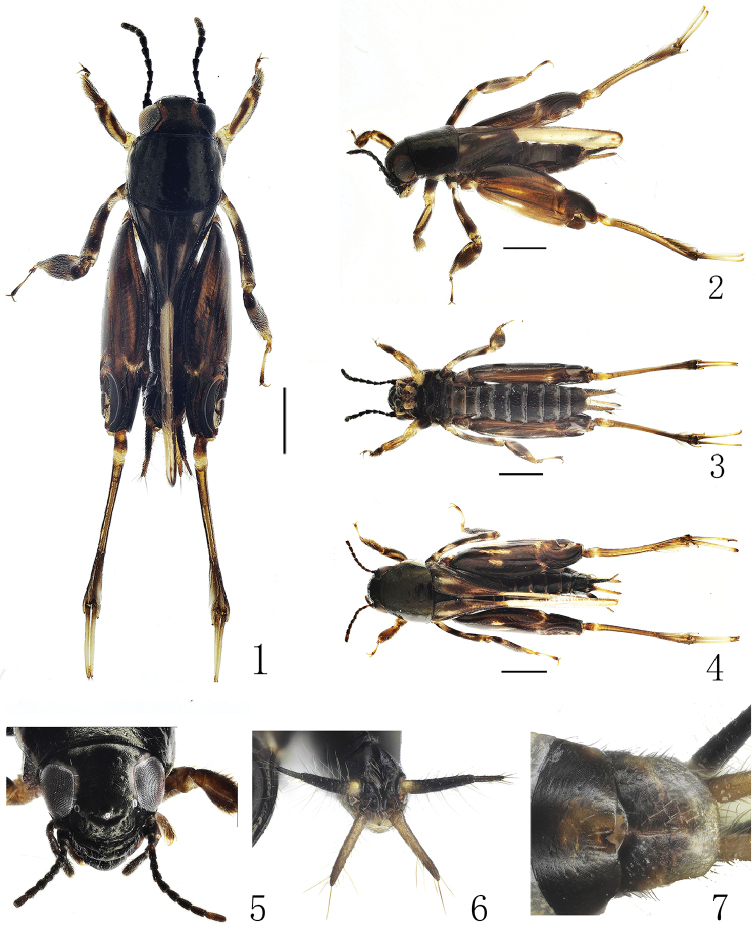
*Xya xishangbanna* sp. nov. **1** body in dorsal view ♂ **2** body in lateral view ♂ **3** body in ventral view ♂ **4** body in dorsal view♀ **5** head in frontal view ♀ **6** end of abdomen in posterior view ♂ **7** gonopore in ventral view ♀. Scale bars: 1.0 mm.

#### Description.

**Male.** Habitus with bright or shiny surface. Head black with brown band along inner margin of compound eyes. Antennae moniliform, black, 10 segments, length of each antennomere almost equal to width. Compound eyes dark brown to black, 2 times broader than longer, rounded in front. Ocelli grayish white. Gena black.

Thorax. Pronotum black, width about 1.25 times length, yellow on ventral margin. Forewings blackish-brown, with pair of basal and medial brown spots. Hindwings yellowish-white, extending beyond the end of abdomen distinctly. Fore and mid legs dark brown with yellow spots. Hind legs with femora dark brown, dorsal margin black, with yellowish-white spots; hind tibiae yellowish-brown, with three (inside) and four (outside) pairs of articulated lamellae.

Abdomen. Abdomen black, gray along posterior margin of each segment. Apex of every sternite with distinct transverse white stripe. Cerci black, paraproctal lobe slightly lighter in coloration than cerci. Epiproct with bottom of the middle “v-shaped” crack in the upper part shallow, and sides straight (Fig. 13A). **Female.** Body larger than male in size. Abdominal segments black with posterior margin gray for each segment. Epiproct rounded. Subgenital plate margin with a notch. Others same as male.

#### Measurement (mm).

Length of body: ♂ 5.63, ♀ 7.16. Length of fore wing: ♂ 1.59, ♀ 2.37. Length of hind wing: ♂ 4.84, ♀ 6.28. Length of hind femur: ♂ 3.76, ♀ 4.50.

#### Distribution.

China (Yunnan).

#### Diagnosis.

This species can be diagnosed by the shiny dark brown hind femora. It is most similar to *X. leshanensis* Cao et al. in the compound eyes with a narrow band along the inner margin. It can be distinguished from the latter by the body with dorsal surface not rough, but more shiny; the compound eyes with a prominent brown band along their inner margin on both sides; the hind femora dark brown, with a pair of white and yellow longitudinal spots; forewings with a pair of basal and apical brownish spots; the length of hind wing more than 4.0 mm; and the epiproct with shallow bottom of the middle “v-shaped” crack in the upper part, and straight sides. In *X. leshanensis*, the body surface is rough; the compound eyes bear a yellowish-white band along the inner margin; the hind femora are black, bearing four yellowish-white spots near the middle; the forewings have no spot; the length of hind wings is less than 4.0 mm; and the epiproct has deep “v-shaped” crack in the upper part with the sides curved (Fig. 13B). Major differences are listed in Table 1.

**Table 1.** Comparison of *Xya xishangbanna* sp. nov. and *Xya leshanensis* Cao et al.

**Characters**	***Xya xishangbanna* sp. nov.**	***X. leshanensis***
Body surface	Not rough, more shiny	Rough, without shiny appearance
Antennomere of antennae	Apical part narrower than basal part in width	Apical part almost same as basal part in width
Compound eyes	With brown band along inner margin	With yellowish-white band along inner margin
Hind femora	Dark brown, with pair of white and yellow longitudinal spots	Black, with four yellowish-white spots near the middle
Forewings	Blackish-brown with a pair of basal and apical brownish spots, more than 1.4 mm long	Blackish-brown without spots, less than 1.4 mm long
Hindwings	More than 4.0 mm long	Less than 4.0 mm long
Epiproct	Bottom of the middle “v-shaped” crack in the upper part is shallow, and the sides straight.	Bottom of the middle “v-shaped” crack in the upper part is deep, and the sides curved.

#### Etymology.

The specific epithet is named after Xishuangbanna, the type locality.

### Xya yunnanensis sp. nov.

Taxon classificationAnimaliaOrthopteraTridactylidae

A677092C-E40F-583D-B9B7-6B309AE2E5C8

http://zoobank.org/0D105287-AD35-420C-A732-F982405D669E

Figures 8–12

#### Type material.

***Holotype***: China • ♂; Yunnan Province, Xishuangbanna, Mengla County, ♂, Wujiazhai; 22.05N, 100.89E; 21–24 Mar. 2019; leg. Chao Tong and Shenzhi Chen.

**Figures 8–12.**
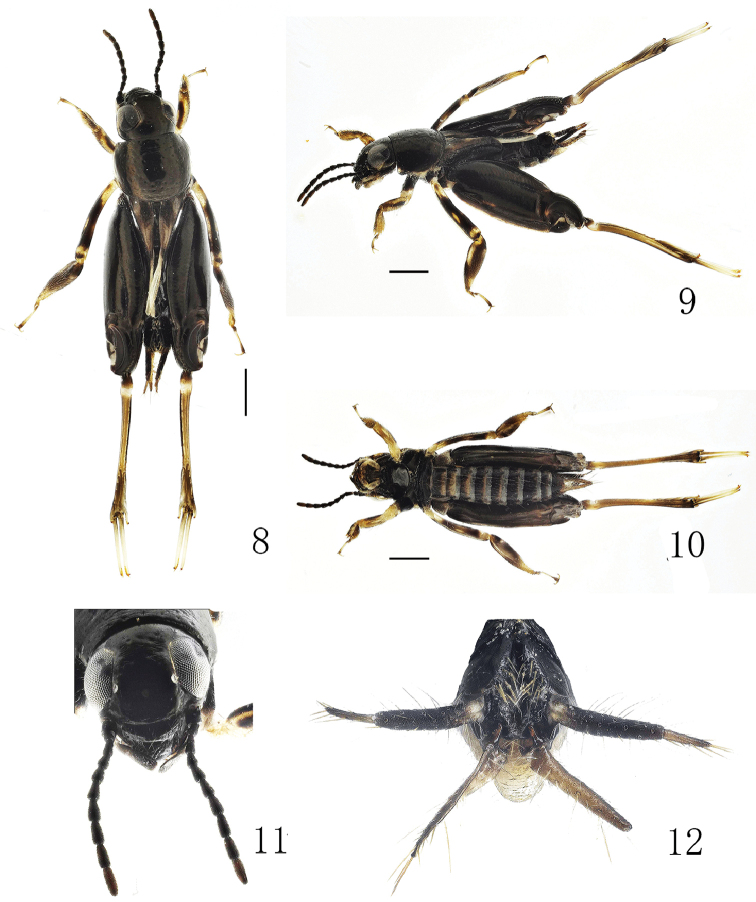
*Xya yunnanensis* sp. nov. **8** Body in dorsal view ♂ **9** Body in lateral view ♂ **10** Body in ventral view ♂ **11** Head in frontal view ♂ **12** End of abdomen in posterior view ♂. Scale bars: 1.0 mm.

#### Description.

**Male.** Head black, without band along inner margin of compound eye. Labial palpi black. Antennae filiform, black, 10 segmented, 10th segment dark fuscous, each segment widens from base to apex. Compound eyes grayish black. Three white ocelli. Gena below the compound eye black.

Thorax. Pronotum black, width about 1.2 times length, with reddish brown luster, white on lateral margin intermittently. Forewings black, with two obscure dirty white sub-rectangular patches at base and apex respectively. Hindwings white, black along posterior margin, about 5/6 length of abdomen. Fore legs yellowish-white; femora with black longitudinal stripe; tarsi with three yellowish-white distal spines. Mid legs black, with yellowish-white irregular markings on femora and tibiae. Hind legs with femora black, with a narrow yellowish-brown marking on basal 1/3 ventrally; semi-lunar process black, yellowish-brown at base; tibia yellowish-brown, darkens toward apex, with three (inside) and four (outside) pairs of articulated lamellae.

**Figure 13.**
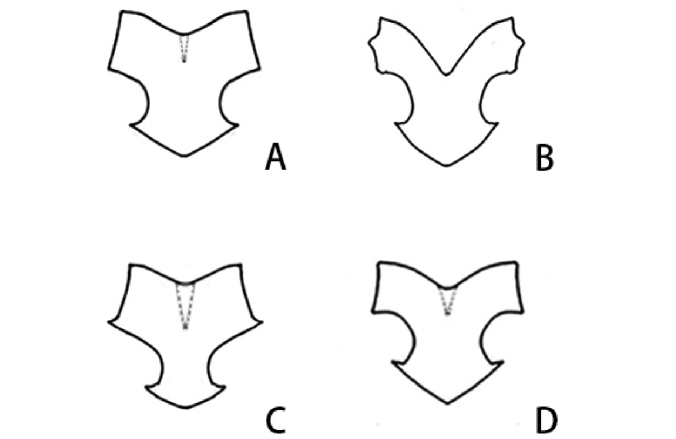
The line diagrams for male epiproct of four *Xya* species. **A***Xya xishangbanna* sp. nov. **B***Xya leshanensis***C***X. yunnanensis* sp. nov. **D***X. sichuanensis*.

Abdomen. Abdomen black, white along posterior margin of each segment. Cerci with two segments, 1st segment black, white at base; 2nd segment pale fuscous, with sparse long white setae. Stylus black on outer side, pale fuscous on inner side, shorter than cerci. Epiproct with shallow “v-shaped” crack in the upper part, bottom of the side edge has a sharply angled protrusion, and width of the narrow lower anchor-shaped base is less than 1/2 the width of the upper one (Fig. 13C).

#### Female.

Unknown.

#### Measurement (mm).

Length of body: ♂ 5.43. Length of fore wing: ♂ 1.28. Length of hind wing: ♂ 2.47. Length of hind femur: ♂ 3.72.

#### Distribution.

China (Yunnan).

#### Diagnosis.

This species can be diagnosed by the compound eye bearing no narrow band along the inner margin. It is most similar to *X. sichuanensis* Cao et al. in having four markings on the forewing, and lacking a patch on the pronotum dorsally. It can be distinguished from the latter by the compound eyes without a narrow band along the inner margin; with no ring around the median ocelli; the black gena below the compound eye; the forewing with obscure dirty white sub-rectangular patches, the length of fore wing about 1.28 mm; the white hindwing; and the epiproct with bottom of the side edge with a sharply angled protrusion and the narrow lower anchor-shaped base less than 1/2 the width of the upper one. In *X. sichuanensis*, the compound eyes bear a narrow yellow band along inner margin; bears a yellow ring around the median ocelli; the gena below the compound eye is yellow; the forewings have yellow triangular patches, the length of fore wing is about 0.9–1.1 mm; the hindwings are yellow; the epiproct with bottom of the side edge without a sharply angled protrusion and the large lower anchor base about 4/5 the width of the upper one (Fig. 13D). Major differences are listed in Table 2.

**Table 2.** Comparison of *X. yunnanensis* sp. nov. and *X. sichuanensis* Cao et al.

Characters	*X. yunnanensis* sp. nov.	*X. sichuanensis*
Compound eyes	Without narrow band along inner margin	With a narrow yellow band along inner margin
Median ocelli	Without ring around	With a yellow ring around
Gena below the compound eye	Yellow	Black
Forewings	With obscure dirty white sub-rectangular patches, about 1.28 mm	With yellow triangular patches, about 0.9-1.1 mm
Hindwings	White	Yellow
Hind femora	With a narrow yellowish-brown marking on basal 1/3 ventrally	Without marking on basal 1/3 ventrally
Epiproct	Bottom of the side edge with a sharply angled protrusion and the narrow lower anchor-shaped base less than 1/2 the width of the upper one	Bottom of the side edge without a sharply angled protrusion and the large lower anchor base about 4/5 the width of the upper one

#### Etymology.

The specific epithet is named after Yunnan, the type locality.

#### Biology.

**Figure 14.**
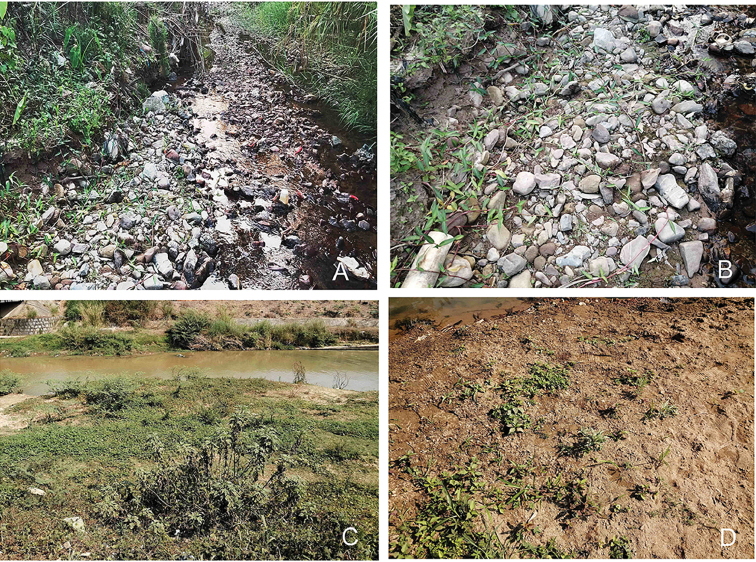
Landscape of habitat in Wuxiangguangchang (**A**, **B**) and in Wujiazhai (**C**, **D**) in Xishuangbanna, Yunnan, China.

These two new species are found along waterways and under mud and stones amidst many different plants and shrubs (Fig. 14). They seem to be living near humid sand with water nearby. The adults were collected during the month of August. They can jump from both the ground and water.

## Key to all *Xya* species in China based on the male superficial characters

**Table:** 

1	Hind femur with marking	**2**
–	Hind femur without marking	**8**
2	Hindwing white	**3**
–	Hindwing black or dark	**7**
3	Antenna with apical 2 or 3 segments white	***X. apicicornis***
–	Antenna with apical 2 or 3 segments black	**4**
4	Forewing with marking	**5**
–	Forewing without marking	***X. leshanensis***
5	Hind femur with pair of longitudinal spots	**6**
–	Hind femur with four spots	***X. riparia***
6	Pronotum with two yellow spots near anterior margin	***X. fujianensis***
–	Pronotum without spots near anterior margin	***X. xishangbanna* sp. nov.**
7	Hind femur with a yellowish-white sub-ovate spot	***X. shandongensis***
–	Hind femur with a white triangular spot	***X. nitobei***
8	Forewing with marking	**9**
–	Forewing without marking	**10**
9	Compound eye with narrow band along inner margin	***X. sichuanensis***
–	Compound eye without narrow band along inner margin	***X. yunnanensis* sp. nov.**
10	Hindwing black	***X. japonica***
–	Hindwing pale yellowish-brown	***X. manchurei***

## Supplementary Material

XML Treatment for Xya xishangbanna

XML Treatment for Xya yunnanensis
